# Anticancer Effect of Deuterium Depleted Water - Redox Disbalance Leads to Oxidative Stress*[Fn FN2]

**DOI:** 10.1074/mcp.RA119.001455

**Published:** 2019-09-13

**Authors:** Xuepei Zhang, Massimiliano Gaetani, Alexey Chernobrovkin, Roman A. Zubarev

**Affiliations:** Division of Physiological Chemistry I, Department of Medical Biochemistry and Biophysics, Karolinska Institutet, SE-17 165 Stockholm, Sweden

**Keywords:** Chemoproteomics, quantification, tandem mass spectrometry, cancer therapeutics, oxidative stress, anticancer, deuterium depleted water, proteomics, redox disbalance

## Abstract

Despite the convincing empirical evidence that deuterium depleted water (DDW, 25–125 ppm deuterium) has anticancer effect, the molecular mechanism remains unclear. Here, redox proteomics investigation of the DDW action in A549 cells revealed an increased level of oxidative stress, whereas expression proteomics in combination with thermal profiling uncovered crucial role of mitochondrial proteins. In the proposed scenario, reversal of the normally positive deuterium gradient across the inner membrane leads to an increased export of protons from the matrix to intermembrane space and an increase in the mitochondrial membrane potential, enhancing the production of reactive oxygen species (ROS). The resulting oxidative stress leads to slower growth and can induce apoptosis. However, further deuterium depletion in ambient water triggers a feedback mechanism, which leads to restoration of the redox equilibrium and resumed growth. The DDW-induced oxidative stress, verified by traditional biochemical assays, may be helpful as an adjuvant to ROS-inducing anticancer therapy.

## Abstract (precis)

The antiproliferation effect of deuterium depleted water (DDW) is well documented, but the mechanism remained elusive. Here three complementary proteomics approaches applied to A549 cells revealed a disbalance brought about by DDW in mitochondria between ROS production and neutralization, thus leading to oxidative stress in the cells. Subsequent validation by orthogonal approaches supported this scenario. Therefore, DDW has potential as an adjuvant in antitumor therapy, especially in the modalities inducing oxidative stress in cancer cells.

## Graphical Abstract

**Figure:**
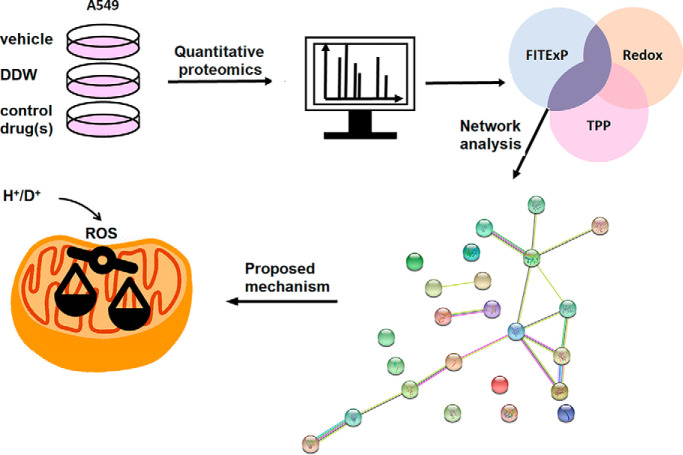

- The antiproliferation effect of deuterium depleted water (DDW) was confirmed in a cell model.
- DDW inhibits cell proliferation through causing a ROS disbalance in mitochondria.
- DDW has a potential as an adjuvant in antitumor therapy.

Deuterium was discovered in 1931 by Harold C. Urey, for which he was awarded the Nobel Prize in Chemistry (1, 2). It has almost immediately become known that the two stable isotopes of hydrogen, protium (H) and deuterium (D), differ not only in their physical parameters (3), but also in chemical (4) and biological (1) properties.

The concentration of D in normal ocean water is 150 ppm, but the deuterium content in natural terrestrial water and ice ranges from 90 to 160 ppm (2, 5). Heavy water (D_2_O) has higher boiling temperature than H_2_O, and thus multiple distillations with early fraction collection can yield DDW, which can contain as little as 1 ppm D (6). A 1993 study found that deuterium depleted water (DDW)^1^ significantly suppresses the growth of L929 fibroblast cells *in vitro*, and inhibits tumor growth in xenotransplanted mice (7). This pioneering research has been followed by many studies investigating potential of DDW in treating cancer (8–19) (Table I). Even though not every study confirmed the DDW effect in cancer (11, 16–17), the aggregate experimental evidence appears to be overwhelmingly in support of the DDW anti-proliferation activity (Table I). But despite extensive research efforts over a quarter of century, no widely accepted molecular mechanism of DDW anticancer action has emerged, although all proposed tentative mechanisms involve the D/H isotope effect that somehow influences cellular processes, such as cell cycle, and/or induces apoptosis (20).

**Table I:** Summary of literature references dealing with anticancer effect of DDW

Organism	Tissue or cell line	Cancer type	DDW range (ppm)	DDW max. of effect and the concentration	Ref.
Mice	L929	Normal fibroblasts	30–600	20% decrease in cell count at 30 ppm; 35% increase in cell count at 600 ppm	(7)
Human	MDA-MB-231	Breast	30	83% increase in survival of xenotransplanted mice	
	MCF7			43% increase in survival of xenotransplanted mice	
Human	PC-3	Prostate	90	15% decrease in cell count	(8)
	MCF 7	Breast		10% decrease in cell count	
	M14	Melanoma		16% decrease in cell count	
	PC3	Prostate	90–95	5% decrease in tumor volume	
			98	2 times higher in apoptosis of cells xenotransplanted in mice	
Mice	A4	Hematopoietic stem	90	42% decrease in cell count	
	IL-3-deprived A4			20% decrease in cell count	
Dog	Tissue	Breast	90–95	67% decrease in tumor volume	
Mice	Tissue	Liver	52	67% increase in H_2_O_2_ generation at mitochondria	(9)
Human	Tissue	Lung	25–105^a^	Noticeable increase in survival time of all 4 lung cancer patients	(10)
Human	A549	Lung	25–150	31% decrease in cell count at 105 ppm	(11)
	H460	Lung		30% decrease in tumor growth of H460 xenograft model mice at 50 ppm	
	HLF-1	Normal embryonic lung fibroblasts		No significant effect	
Human	Tissue	Prostate	85^a^	50% decrease in prostate specific antigen (PSA); 59% decrease in tumor volume; 33% increase in patient survival	(12)
Human	Tissue	Lung	25–105^a^	11% increase in patient survival	(13)
Mice	Tissue	Lung	25	Significant (*p* < 0.05) increase in expression of Kras, Bcl2, Myc	
Human	Tissue	Breast	65–105^a^	2–3 times longer median survival time (MST) compared to only conventional therapy; 3 times longer MST of patients who took DDW more than once than those who took it only once	(14)
Human	CNE-1	Nasopharyngeal	50–150	40% decrease in cell count at 50 ppm	(15)
	MC3T3-E1	Normal preosteoblast		73% increase in cell count at 75 ppm	
Human	MDA-MB-231	Breast	40–150	No significant effect on either tumor or normal cells	(16)
	HCT-116	Colon			
	PC-3	Prostate			
	U-87MG	Glioblastoma multiform			
	AGS	Stomach			
	HDF-1	Normal dermal fibroblast			
Human	A549	Lung	<150^b^	<10% effect on cell apoptosis, not significant	(17)
	ZR-75-1	Breast			
	HT-29	Colon			
Mice	Tissue	Liver	46	35% increase in generation of hydrogen peroxide at mitochondria	(18)
Mice	Tissue	Liver	46	18% decrease in weight of rats; 15% increase in aspartate aminotransferase; 43% increase in (AST) alanine amino-transferase (ALT); 35% increase in generation of hydrogen peroxide at mitochondria	(19)

^a^ In addition to conventional therapies.

^b^ The authors did not mention the concentration range of DDW.

DDW effects on cell growth are not unfeasible, as normal deuterium content corresponds to a rather high deuterium concentration. Indeed, the molar mass of H_2_O is 18 g, and thus 1 L contains 55.6 moles of water, corresponding to a molar concentration of 55.6 m. Considering D_2_O a small molecule additive, the molar concentration for 150 ppm D is 8.3 mm. For comparison, the intracellular concentration of Ca^2+^ is roughly 100 nm, *i.e.* five orders of magnitude lower, although Ca^2+^concentration can increase 10–100 times during various cellular events (21). The intracellular concentration of another important ion, Cl^−^, is ≈4 mm, *i.e.* half of the normal deuterium concentration.

In addition, deuterium is not only different from protons by mass, but also in terms of biologically important properties. The biggest isotope effect in biology is found in NADPH, in which deuterium ion is discriminated compared with proton by up to 60% (22, 23). Such an extreme discrimination level indicates that the D/H balance is biologically important. Indeed, the suppression of growth of different organisms by higher than normal deuterium content is well documented. D_2_O is known to be toxic for higher organisms at ≥25% concentration, but even a <1% D concentration can have a measurable effect on bacterial growth (24).

In the beginning of DDW exploration, a paradigm has emerged that deuterium is essential for cell proliferation (7). This paradigm is sometimes repeated even today; yet there are several facts that do not fit in it. One such fact is that far from all cancer cells, including some aggressive types, are sensitive to DDW (8, 11). Another unexplained fact is that the DDW toxicity for cancerous cells does not increase monotonously with deuterium depletion, often reaching maximum at relatively modest values between 50 and 105 ppm D (11, 15). Although varying effect on different cancer types is a common phenomenon for conventional anticancer drugs, a toxicity decrease with increased concentration (analog of deuterium depletion) is extremely rare, if not unprecedented.

To shed light on these intriguing questions, we decided to study the DDW anticancer function using recently developed chemical proteomics strategies. For this purpose, we treated DDW as a chemical anticancer agent, and contrasted its action at the protein level with that induced by several common anticancer molecules at concentrations that suppress cell growth to a similar degree.

## EXPERIMENTAL PROCEDURES

### Experimental Design and Statistical Rationale

**Fig. 1.**
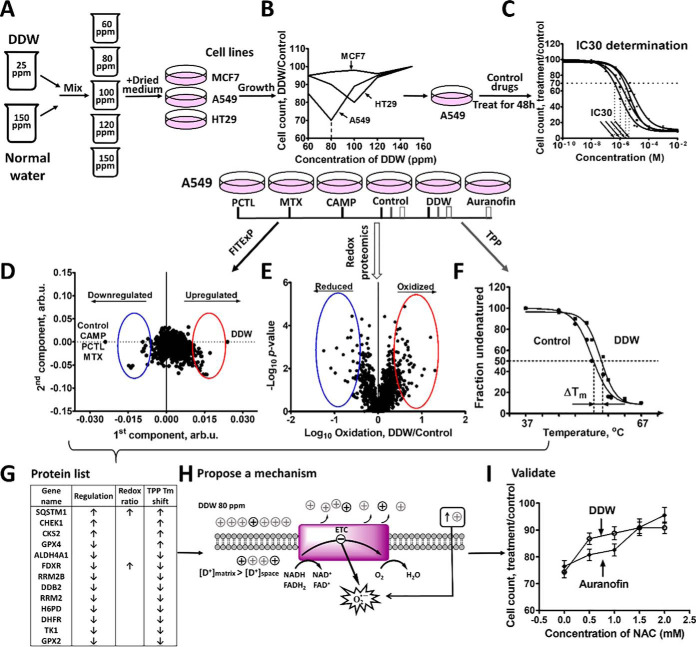
**The layout of the proteomics-based characterization of DDW anticancer mechanism.** *A,* DDW with varying deuterium concentration was prepared by mixing NW (150 ppm D) and 25 ppm DDW in different proportions. MCF 7, A549 and HT29 cells were grown in a DDW medium. *B*, Measurement of the cell lines responses to DDW. *C*, Determination of the concentrations of control drugs that inhibit cell growth by 30 ± 5% (IC_30_). *D*, Identification by FITExP analysis of the most regulated by DDW proteins compared with control drugs and NW. *E*, Measurement by redox proteomics of oxidation-reduction disbalance caused by DDW compared with NW control and auranofin. *F*, Measurement by TPP of proteome stability changes caused by DDW. *G*, Summary of the proteomics results reveals proteins mostly likely involved in DDW action. Proposing a DDW action mechanism (*H*) and its validation by additional experiments (*I*).

The overall study plan is shown in Fig. 1. After selection based on literature data of a preliminary cell panel (MCF7, A549 and HT29 cells), the most sensitive cell line as well as the deuterium concentration of maximum growth suppression in this cell line were to be determined (Fig. 1*A*, 1*B*). For a group of selected control drugs (PCTL, MTX, CAMP and auranofin), the concentration inducing the same growth suppression over 48 h as DDW was to be identified (Fig. 1*C*). Comparing the proteome data on the sensitive cells treated with DDW with the same cells treated with drugs to the same degree of growth suppression would identify the proteins specifically up- and downregulated by DDW. This method is called Functional Identification of Targets by Expression Proteomics (FITExP) (27–31). Fig. 1*D* illustrates how OPLS-DA, a supervised variant of PCA, identifies the specifically up- and downregulated proteins. In parallel, redox proteomics of DDW- and auranofin-treated cells, as well as controls, would uncover changes in the oxidative states of thiols in cellular proteins (Fig. 1*E*). TPP (25, 26) was planned to reveal which variations observed by FITExP and redox proteomics were accompanied by changes in the thermal stability of proteins (Fig. 1*F*). Comparison of the results from these three complementary proteomics techniques would identify most affected proteins (Fig. 1*G*), which could lead to formulation of a DDW mechanistic hypothesis (Fig. 1*H*). This hypothesis would then be tested by additional experiments, including traditional biochemical assays (Fig. 1*I*).

Following the above plan, the proteomics data were obtained by nanoLC-MS/MS in four experimental parts. In order to make appropriate statistical analysis, all treatments were performed in biological replicates. The number of replicates was selected based on the desired statistical power in a particular analysis part: 4 for protein abundances analysis (part 1), 2 for time course analysis (part 2), 3 for redox proteomics analysis (part 3) and 2 for TPP analysis (part 4). Each set of TMT10 labeling samples were combined and fractionated into 10 fractions. In total, 113 TMT labeled samples including 11 sets of TMT10 labeled and 3 sets of iodoTMT6 labeled samples were analyzed. NanoLC-MS/MS experiments were performed with a 120 min LC gradient. In each part of the experiment, separate controls treated with the vehicle were included. Samples of each part were analyzed on LC-MS in random order to reduce the “order of injection” effect. In part 1, A549 cells were treated with CAMP, MTX, PCTL or grown in 80 ppm DDW (20 samples). In part 2, the cells were grown in 80 ppm DDW, or treated with MTX and PCTL for 4–48 h (5 time points, 40 samples). In addition, 2 control samples treated with the vehicle for 48 h were included in each set of TMT10 labeled samples for normalization. In part 3, the cells were grown in 80 ppm DDW or treated with auranofin (3 samples). Finally, in part 4, the cells were grown in 80 ppm DDW and afterwards incubated at 10 temperature points from 37 to 67 °C, followed by centrifugation and digestion of the supernatant (40 samples). Quality check was performed by calculating the variation (CV) between the replicates as well as by building PCA models to verify the small data spread between the replicates.

### Cell Lines and Cell Survival Measurement

MCF7, A549, and HT29 cell lines were obtained from the cell bank at the Karolinska Institutet. Cells were grown in DEME (11685260, Thermo Fisher Scientific, Waltham, MA) supplemented with 10% heat-inactivated FBS (11560636, Thermo Fisher Scientific), 1% penicillin/streptomycin (15140–122, Gibco, Waltham, MA) and 2 mm l-Glutamine (17–605E, Fisher Scientific) in a humidified atmosphere containing 5% CO_2_ at 37 °C. DDW of different concentrations were prepared by mixing 25 ppm DDW (obtained from G. Somlyai at HYD LLC, Hungary) and normal Millipore water (NW). First, the mixed water was shaken for 48 h for complete mixing. Second, the water was heated to 70 °C and kept for 0.5 h followed by cooling to RT. After repeating the second step for additional 4 times, the blended water was used to prepare culture medium by dissolving DMEM-high glucose powder (D5648, Sigma, Virginia Beach, VA), 3.7 g/L sodium bicarbonate (S5761, Sigma), 10% FBS (v/v) and 1% penicillin/streptomycin (v/v). Cells were washed by DDW medium twice before seeding. In order to avoid cell overgrowth, seeding numbers were selected according to growth, size and volume of different cells in the survival measurement. 5000 cells for A549, 10,000 cells for MCF7 and HT29 were seeded in each well of 96-well plate. After 48 h, 5 mg/ml MTT (M6494, Thermo Fisher Scientific) in PBS buffer (17–516F, Lonza) was added in each well and the plate was incubated at 37 °C for 4 h. Then the cells were incubated at 37 °C for 14 h with 0.1 g/ml SDS (H5113, Promega, Madison, WI) in 0.1 m HCl to dissolve formazan formed after adding MTT. The absorbance of formazan in each well was measured using Epoch microplate spectrophotometer (Biotek, Vicenza, Italy) at 570 nm. The cell survival percentage for each treatment and control was calculated, with inhibitory curve analysis being performed using the software Prism v. 5.02 (GraphPad, San Diego, CA).

### Cell Growth Inhibition by Control Drugs

The control drugs CAMP (C9911, Sigma), MTX (M9929, Sigma), PCTL (T7191, Sigma), and auranofin (A6733, Sigma) were used to treat cells at different concentrations. 5000 A549 cells were seeded in each well of a 96-well plate for 48 h. After that, the inhibitory curves were obtained as described above. The IC_30_ concentrations at which cell proliferation was inhibited by 30 ± 5% were used in further experiments. For combined effect of auranofin and 80 ppm DDW, A549 cells were washed with 80 ppm DDW medium and then 5000 cells both in 80 ppm DDW and NW medium were seeded in each well of a 96-well plate. 5 μm, 4 μm, 3 μm, 2 μm auranofin solution and 1% DMSO solution were used to treat the cells grown in DDW or NW medium for 48 h. For NAC (A7250, Sigma) experiments, 5000 cells both in 80 ppm DDW and NW medium were seeded in each well of a 96-well plate. Auranofin was added into each NW medium well to a 3 μm concentration. Afterward, NAC was added at 2 mm, 1.5 mm, 1 mm, 0.5 mm, 0 mm (NW) concentrations, and cells were incubated for 48 h, followed by cell counting.

### Measurement of Cellular ROS Concentration

Five thousand A549 cells were seeded in each well of a 96-well plate in four replicates. After incubation overnight, the cells were washed with NW or DDW (same deuterium concentration as in the growth media) three times. As a positive control for oxidative stress, 3 μm auranofin was used. The combined effect of auranofin and DDW on ROS production was measured by treating the cells with 3 μm auranofin and/or DDW medium. After 24 h treatment, the cells were washed with PBS twice and 100 μl of 20 μm DCF-DA in PBS was added into each well. After 30 min incubation at 37 °C in darkness, the fluorescence intensity was measured by Infinite^®^ M200 PRO (TECAN, Männedorf, Switzerland). The excitation and emission wavelengths were 485 nm and 535 nm, respectively. In parallel, the relative cell survival was measured by the MTT assay described above. The fluorescence intensity in each well was normalized to the average intensity of each alive cell. The time course analysis of ROS production was performed by measuring ROS level in cells growing in different growth conditions at several time points.

### FITExP Sample Preparation

Fifteen thousand A549 cells were seeded in each well of a 6-well plate and the cells were grown in 80 ppm DDW medium or treated with, 0.5 μm CAMP, 2 nm PCTL or 0.5 μm MTX in four replicates. After 48 h treatment, the cells were collected and lysed using 50 mm Tris (741883, Sigma) buffer and 8 m urea (U5378, Sigma), 1% SDS, and protease inhibitor (5892791001, Sigma) at pH 8.5. For the time course experiment, the cells were grown in either NW or 80 ppm DDW and treated with either MTX or PCTL for 4, 15, 26, 38, and 48 h. After protein reduction using 8 mm DTT (10708984001, Sigma) and alkylation using 25 mm IAA (I1149, Sigma), the proteins were precipitated using cold acetone at −20 °C overnight followed by centrifugation and resuspension. Proteins were then digested by Lys C (125–05061, Wako Chemicals GmbH, Neuss, Germany) (1:75 enzyme to protein ratio) at 30 °C for 6 h and trypsin (V5111, Promega) (1:50 enzyme to protein ratio) at 37 °C overnight. After labeling using the TMT-10 reagent (90110, Thermo Fisher Scientific), desalting on C18 Sep-pak columns (WAT054960, Waters, Milford, MA) and fractionation using high pH reversed-phase peptide fractionation kit (84868, Thermo Fisher Scientific) according to manufacturer's instructions, the obtained 10 fractions of peptides in each sample were analyzed by shotgun proteomics using nanoLC-MS/MS.

### Redox Proteomics Sample Preparation

Fifteen thousand A549 cells were seeded in each well of a 6-well plate and the cells were grown in 80 ppm DDW or NW, and treated with either a vehicle or auranofin at 3 μm in 3 replicates. After 48 h, the cells were collected and lysed in lysing buffer at pH 8.0: 50 mm HEPES (U5378, Sigma) with addition of 8 m urea, 1 mm EDTA (E9884, Sigma), 1% SDS and protease inhibitor. The samples were incubated with 4.4 mmol/L of iodoTMT-126, iodoTMT-127 and iodoTMT-128 (90102, Thermo Fischer Scientific) over night at 37 °C. Free SH and SSH groups were blocked in this stage. After precipitation using methanol/chloroform, the samples were dissolved in 50 mm HEPES buffer including 8 m urea. 10 mm TCEP (T2556, Thermo Fischer Scientific) was used to reduce disulfides at 50 °C for 1 h. After precipitation followed by resuspension, the samples were labeled by iodoTMT-129, iodoTMT-130 and iodoTMT-131 reagents overnight at 37 °C. Afterward, the labeled samples were precipitated and then resuspended for digestion using Lys C (1:75, enzyme to protein ratio) at 30 °C for 6 h and trypsin (1:50, enzyme to protein ratio) at 37 °C overnight. After desalting, the iodoTMT-labeled peptides were enriched using immobilized anti-TMT resin (90076, Thermo Fischer Scientific) according to manufacturer's instructions. The enriched peptides were analyzed by shotgun proteomics using nanoLC-MS/MS.

### TPP Sample Preparation

Three million cells were seeded in a T-175 flask and grown in either 80 ppm DDW or NW medium in 2 replicates. After 48 h, the cells in each flask were collected, washed and divided into ten aliquots. These were incubated for 3 min at the following ten temperature points: 37, 41, 44, 47, 50, 53, 56, 59, 63, and 67 °C. After cells were kept at RT for 5min to cool down, the cells were lysed by three freeze/thaw cycles. After ultracentrifugation at 35,000 rpm/min at 4 °C for 30 min, the supernatant was collected, reduced with 8 mm DTT and alkylated with 25 mm IAA. The samples were precipitated using cold acetone at −20 °C overnight, digested by Lys C (1:75, enzyme to protein ratio) for 6 h at 30 °C and then by trypsin (1:50, enzyme to protein ratio) overnight. After labeling using TMT-10 reagent, desalting with C18 Sep-pak columns and fractionation by a high pH kit as above, the 10 fractions of peptides for each sample were analyzed by nanoLC-MS/MS.

### NanoLC-MS/MS Analysis

NanoLC-MS/MS analyses of FITExP and TPP samples were performed on a Q Exactive HF mass spectrometer, and of redox proteomics samples—on an Orbitrap Elite mass spectrometer (both - Thermo Scientific). The nanoLC system was an UltiMate 3000 (Thermo Scientific). FITExP and TPP samples were pre-concentrated and desalted online using a PepMap C18 nano trap column (length, 2 cm; inner diameter, 75 μm; particle size, 3 μm; pore size, 100Å; Thermo Scientific) at a flow rate of 3 μl/min for 5 min. Peptide separation was performed using an EASY-Spray C18 reversed-phase nano LC column (Acclaim PepMap RSLC; length, 50 cm; inner diameter, 2 μm; particle size, 2 μm; pore size, 100 Å; Thermo Scientific) at 55 °C and a flow rate of 3 μl/min. Peptides were separated using a binary solvent system consisting of 0.1% (v/v) FA, 2% (v/v) ACN (solvent A) and 98% ACN (v/v), 0.1% (v/v) FA (solvent B). They were eluted with a gradient of 4–26% B in 120 min, 26–95% B in 10 min. Subsequently, the analytical column was washed with 95% B for 5 min before re-equilibration with 4% B. The first step of gradient was split into two parts, 4–10% B in 100 min plus 10–26% B in 20 min for fraction 3 to 5, and 4–14% in 100 min plus 14–26% B in 20 min for fractions 6 to 8. Mass spectra were acquired in a mass-to-charge (m/z) range of 375–1500 with a resolution of 120,000 at *m*/*z* 200. The AGC target was set to 3 × 10^6^ with a maximum injection time of 100 ms. The 17 most intense peptide peaks were selected for peptide fragmentation via HCD with the NCE value set at 33. The ion selection abundance threshold was set at 0.1% with charge exclusion of z = 1 ions. The MS/MS spectra were acquired at a resolution of 60,000, with a target value of 2 × 10^5^ ions or a maximum injection time of 120 ms. The fixed first *m*/*z* was 100, and the isolation window was 1.2 *m*/*z*. The instrument was operated in the positive ion mode for data-dependent acquisition of MS/MS spectra with a dynamic exclusion time of previously selected precursor ions of 30 s. For redox samples, peptides were washed and pre-concentrated using C18 reversed-phase nano LC column (Acclaim PepMap RSLC column; length, 50 cm; inner diameter, 75 μm; particle size, 2 μm; pore size, 100 Å; Thermo Scientific) at 35 °C and a flow rate of 3 μl/min. Peptides were separated using a binary solvent system consisting of 0.1% (v/v) FA, 2% (v/v) ACN (solvent A) and 98% ACN (v/v), 0.1% (v/v) FA (solvent B). They were eluted with a gradient of 2–26% B in 120 min, 26–95% B in 10 min, and 95–2% B in 10 min. Subsequently, the analytical column was washed with 98% B for 5 min before re-equilibration with 98% A. Mass spectra were acquired in a mass-to-charge (*m*/*z*) range of 375–1200 with a resolution of 120,000 at *m*/*z* 200. The 10 most intense peptide peaks were selected for HCD experiments in the linear ion trap with the following parameters: normalized CE, 35%; activation time, 10 ms; AGC, 5000; maximum injection time, 150 ms; isolation width, *m*/*z* 1.6. The dynamic exclusion time of previously selected precursor ions was set to 45 s and only +2 or higher charged ions were selected for MS/MS, recorded with a resolution of 30,000. The ion selection abundance threshold was set at 0.1% with exclusion of singly charged ions.

### MS Data Analysis

The mass spectrometric raw data were analyzed with the MaxQuant software (version 1.5.6.5). A FDR of 0.01 for proteins and peptides and a minimum peptide length of 6 amino acids were required. The mass accuracy of the precursor ions was improved by the time-dependent recalibration algorithm of MaxQuant. The Andromeda search engine was used to search the MS/MS spectra against the Uniprot human database (UP000005640_9606 and UP000005640_9606_additional, last modified on January 26, 2019) combined with 262 common contaminants and concatenated with the reversed versions of all sequences. Enzyme specificity was set to “Specific Trypsin/P, Lys/P”. No more than two missed cleavages were allowed. Other allowed modifications were cysteine carbamidomethylation (fixed) as well as protein N-terminal acetylation, asparagine and glutamine deamidation and methionine oxidation (variable). A maximum of two missed cleavages were allowed. Peptide identification was based on a database search with an initial mass deviation of the precursor ion of up to 7 ppm. Mass tolerance for precursor ions was 20 ppm on the *m*/*z* scale (initial search) and 4.5 ppm (main search) and the MS/MS mass tolerance was set to 20 ppm on the *m*/*z* scale. Only proteins quantified with at least two peptides were considered for quantification and all the known contaminants were ignored. For redox proteomics data analysis, the data from both treatment and control experiments were searched with MaxQuant 1.5.6.5 as described above; however, no fixed modification was set and protein N-terminal acetylation, asparagine and glutamine deamidation and methionine oxidation were selected as variable modifications. The quantification was based on iodo6plexTMT reporter ions in MS/MS. The option “Match between the runs” was used in each experiment. For each set of TMT-10 labeled samples, MS/MS reporter abundances in each channel were normalized to that of the TMT-126 channel.

### Principal Component Analysis (PCA)

PCA and OPLS-DA were performed using SIMCA 15.0 (Umetrics). Normalized TMT-10 channel abundances were analyzed except for redox proteomics data, for which the oxidation percentage was used. OPLS-DA model performance was reported as cumulative correlation coefficients for the model (*R* ^2^ × [cum]), with predictive performance being based on 7-fold cross-validation calculations (*Q*^2^[cum]) and analysis of variance of cross-validated residuals (CV-ANOVA) *p* values.

### Protein Network and Pathway Analysis

STRING v10.5 was used to map significantly regulated proteins onto protein-protein interaction networks. Gene names corresponding to up- and downregulated proteins were submitted for analysis on the STRING web-site (http://string-db.org). Medium confidence threshold (0.4) was used to define protein-protein interactions. The in-built gene set enrichment analysis with the whole genome background was used to identify enriched gene ontology terms and KEGG pathways.

### Statistical Analysis

Analysis of the quantitative proteomics data for TPP was performed using an in-house developed R library (https://github.com/snp/CETSA). In brief, protein abundances at different temperatures were fitted to a model sigmoid protein melting curve, and the melting temperature Tm was determiend as a middle point of that curve (25, 26).

## RESULTS

### DDW Suppresses Growth of Cancer Cells

**Fig. 2.**
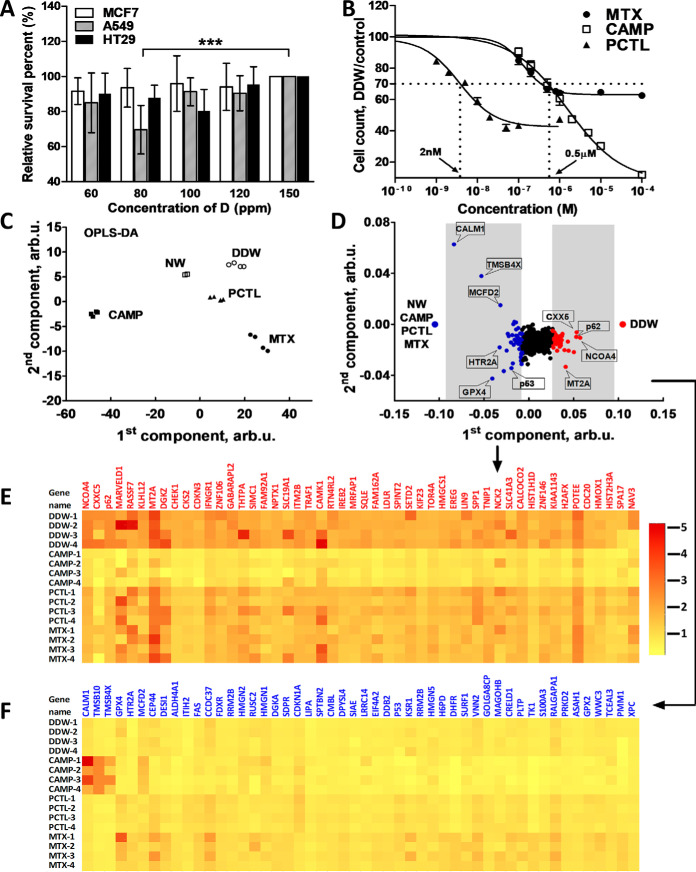
**Determination of the most sensitive to DDW cell line and FITExP analysis at the deuterium concentration of maximum suppression.** *A*, Survival of MCF7, A549 and HT29 cell lines grown in media with different deuterium concentrations. *B*, Inhibition curves of CAMP, PCTL and MTX, and determination of IC_30_. *C*, OPLS-DA of protein abundances for different treatments at IC_30_. *D*, Loading scatter plot of OPLS-DA reveals the top 50 upregulated proteins (red dots) and the top 50 downregulated proteins (blue dots) specific for DDW treatments. (*E, F*) Heat map of top 50 specifically up- (*E*) and downregulated (*F*) proteins in DDW treatment. *A* and *B* show the mean ± SEM of four independent experiments, single measurement, *** *p* < 0.005 in two-way ANOVA with post hoc *t* test.

The DDW effect on proliferation of human breast adenocarcinoma MCF7, human lung carcinoma A549 and human colorectal adenocarcinoma HT29 cells was assessed. When the cells were grown for 48 h in DDW with deuterium concentration of 60 ppm, 80 ppm, 100 ppm, and 120 ppm and in normal water (NW, 150 ppm D) as a control, MCF7 cells responded weakly to DDW treatment, whereas HT29 cells showed a ≈20% reduction in proliferation at 100 ppm D. The A549 cells turned out to be most responsive to DDW (Fig. 2*A*), with 80 ppm DDW inhibiting cell proliferation by 32% (*p* < 0.005). Therefore, the A549 cells were selected for further proteomics studies. At both lower and higher D concentrations compared with maximum suppression, cell proliferation restored to normal levels (supplemental Fig. S1).

### Effect of DDW on Protein Abundances

The underlying assumption in FITExP is that the most regulated proteins in deep apoptosis (24–72 h treatment) are those most intimately related to the action mechanism of the toxic agent. To reach the desired analysis specificity and differentiate the mechanistic proteins from the general-response proteins related to death and survival pathways, control drugs are applied at concentrations inducing similar level of proliferation suppression (27–31). We chose antifolate agents (MTX), tubulin-active antimitotic agents (PCTL), and TOP1 inhibitors (CAMP) as control drugs for DDW because their targets are well known and the FITExP strategy has been applied to these drugs in our previous work (27). The A549 cells were grown in NW control as well as in 80 ppm DDW, 0.5 μm CAMP, 2 nm MTX and 0.5 μm PCTL, which in all cases but control reduced cell count by 30 ± 5% (Fig. 2*B*).

The cellular proteomes were then extracted and analyzed by nanoLC-MS/MS. A total of 6910 proteins were identified and quantified, with 6370 proteins common for all treatments (supplemental Table S1). OPLS-DA (Fig. 2*C*) provided cleared separation of treatments, with the replicates clustering together. When DDW group was contrasted to all other treatments and the control, the most specifically up- and downregulated proteins were identified (Fig. 2*D*). Top 50 up- and downregulated by DDW proteins were selected according to the “VIP predictive value” (Fig. 2*E*, 2*F*; supplemental Table S2), and classified by GO terms as well as KEGG pathways (supplemental Tables S3 and S4). Cell cycle was found as the most enriched process for upregulated proteins, whereas for downregulated proteins, two molecular functions were over-represented: ribonucleoside-diphosphate with thioredoxin disulfide as acceptor and oxidoreductase activity. Mapping the downregulated proteins revealed the p53 signaling as the most deactivated pathway. p53 is a multi-faceted tetrameric transcription factor (32), which regulates more than 2500 genes (33) implicated in cell cycle progression (34, 35), cell death signaling (36, 37), metabolism (38, 39), DNA repair (40), and angiogenesis (41). p53's potent and versatile anti-cancer activity profile, together with genomic and mutational analyses documenting inactivation of p53 in more than 50% of human cancers, determined the critical role p53 plays in anticancer therapy development. Our finding of p53's being one of the most downregulated proteins in DDW treatment is consistent with the previous reports of DDW suppressing the p53 expression in different organs of animals, including the lungs (42).

Another pathway suppressed by DDW was glutathione metabolism. Glutathione accounts for more than 90% of cellular nonprotein thiols (43), participates in establishing cellular redox balance, and thus plays an important role in tumor development and therapy. 10–15% of the total cellular glutathione is found in mitochondria. Glutathione is of paramount importance in protecting the organelle from ROS produced through mitochondrial ETC (44). In various types of tumors, the ratios of oxidized glutathione to reduced glutathione are found to be elevated, which makes neoplastic tissues more resistant to chemotherapy (45). This result hinted that DDW may act through induction of disbalance in the cellular redox equilibrium.

To assess the dynamics of protein regulation by DDW, we performed a time course analysis, in which the abundances of the 50 up- and downregulated proteins (Fig. 2*E*, 2*F*) were analyzed 4–48 h after growth in either NW or in 80 ppm DDW, as well as in NW with addition of control drugs MTX and PCTL (supplemental Table S5–S8). The moment, when the abundance of the most up- or downregulated proteins changed by ≥20% was taken as a characteristic time of action of the respective agent (DDW or control drug). The characteristic time for MTX was 4 h (supplemental Fig. S2*A*), whereas that of PCTL was 38 h (supplemental Fig. S2*B*). For DDW, the regulation of p62 (also known as SQSTM1) was used to measure the characteristic time. The regulation of p62 exceeded 1.2 after 28 h (supplemental Fig. S2*C*). As an alternative way of assessing the characteristic time of inducing proteome changes, the *p* value between the regulations of the top 50 upregulated proteins taken as a group and the group of downregulated proteins was calculated. The statistical significance (*p* < 0.05) was reached at 4 h for MTX, 26 h for PCTL and 15 h for DDW (supplemental Fig. 2*D*). It is known that MTX induces cell apoptosis through arresting the synthesis of DNA, RNA, thymidylates, and proteins (46–48), and thus acts on a relatively short time scale. In contrast, PCTL blocks the progression of mitosis, with prolonged activation of the mitotic checkpoints triggering apoptosis - and hence the long characteristic time of that drug. The characteristic time of DDW action appears to be intermediate. Note that in the STRING analysis, the proteins with most significant abundance changes mapped on the cell cycle KEGG pathways. This hinted that DDW may act through arresting or prolonging the late S and G2 phases in A549 cells.

### DDW Affects Cellular Redox Balance

Oxidative stress can induce severe disruption of signaling pathways and affect cancer cell survival, proliferation, invasion, angiogenesis, apoptosis, and other vital cellular processes (49, 50). Several studies found that oxidative stress is a risk factor for developing different kinds of cancers (51). Oxidative stress is also often considered to be one of the hallmarks of cancer. Yet enhanced oxidative stress is also a venue explored by some modern anticancer agents, including auranofin (52, 53).

**Fig. 3.**
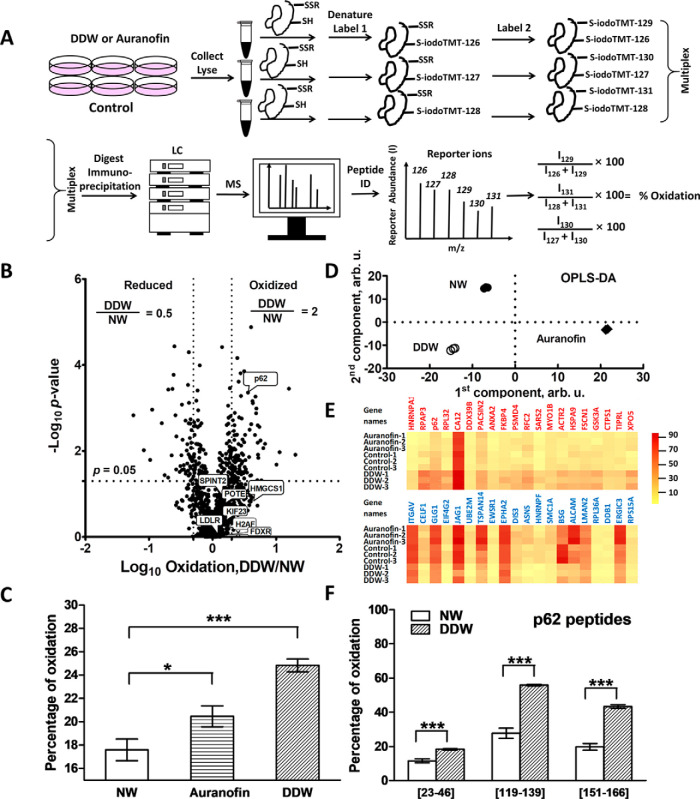
**Redox proteomics analysis of the DDW effect.** *A*, Redox proteomics workflow. *B*, Volcano plot for cells treated with DDW compared with NW. Lines indicate differences of 2 in mean ratios and *p* value of 0.05 in two-tailed unparied *t* test. *C*, Average oxidation levels of cells grown in NW, auranofin and DDW. *D*, OPLS-DA of redox proteomics data. *E*, Heatmap of 20 most oxidized (above) and reduced (below) proteins in cells grown in DDW. *F,* Oxidation levels of p62 peptides in DDW *versus* NW control. *B* shows the mean of three independent experiments. The columns in *C* and *F* show the mean ± SEM in three independent experiments; * *p* < 0.05, *** *p* < 0.005 in two-tailed unpaired *t* test.

To test the hypothesis that oxidative stress is involved in DDW action, we measured the redox effect of DDW on A549 cellular proteins. Redox proteomics is a special analysis type that typically involving the quantification of free cysteine thiol modifications, such as disulfide formation as well as a plethora of other modifications, *e.g.* S-nitrosylation, S-sulfhydration, etc (54–56). The redox proteomics workflow is shown in Fig. 3*A*. Auranofin was selected as a positive control drug, and its IC_30_ in A549 cells was determined (supplemental Fig. S3). Because redox proteomics targets only Cys-containing peptides, it usually produces fewer protein identifications than conventional expression proteomics analysis. Here, 2935 thiol-containing proteins were identified in total (supplemental Table S9–S10). Only proteins with oxidation ratios quantified in all three replicates of all samples were considered for further analysis; this resulted in a shorter list with 609 proteins (Fig. 3*B*). Surprisingly, we observed that DDW increased the overall oxidation level even more than auranofin (Fig. 3*C*). The oxidation percentages determined in control samples were somewhat higher than what has been reported in other studies (57). This could be because of *in vitro* oxidation during sample preparation. However, this phenomenon should not have affected the conclusions in our study, as they were drawn based on the relative changes in oxidation levels. OPLS-DA showed good clustering of the data by treatments (Fig. 3*D*). As in the FITExP case, we contrasted the DDW samples with auranofin treatment pooled into one group with control. The 20 most oxidized and reduced proteins (Fig. 3*E*, supplemental Table S11) were selected according to the “VIP predictive value” in OPLS-DA for further analysis. p62 was one of the most oxidized proteins; its average oxidation level in DDW was 34% compared with 18% for control (*p* < 0.005). In OPLS-DA (supplemental Fig. 2*B*, supplemental Table S14), the p62 peptide (119–139) NMVHPNVICDGCNGPVVGTR was the most oxidized peptide (supplemental Table S12), with 49% average oxidation in DDW compared with 27% oxidation for control. Two other p62 peptides, (23–46) FSFCCSPEPEAEAEAAAGPGPCER and (151–166) CSVCPDYDLCSVCEGK were also found to be oxidized (*p* < 0.005, Fig. 3*F*, supplemental Table S12). The peptides (119–139) and (151–166) are in the zinc finger domain of p62 that confers to this protein the ability to dock with key interacting proteins (58).

As it is usually the case, although most proteins increased their oxidative level, some proteins have decreased it (Fig. 3*B*, supplemental Table S13).

### DDW Effect on Proteome Thermal Stability

**Fig. 4.**
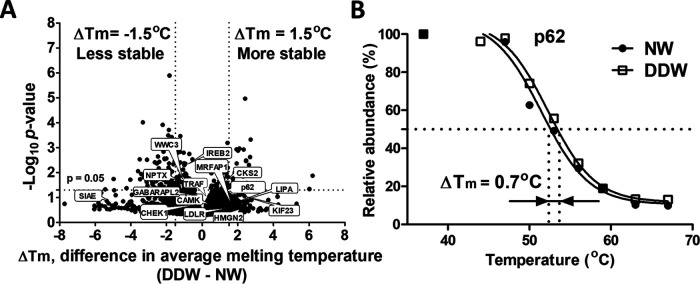
**Changes in thermal stability of proteins upon DDW treatment.** *A*, Volcano plot of melting temperature shifts compared with NW control. Lines indicate a difference in average melting temperature of ≥ 1.5 °C and *p* value of 0.05 (-Log_10_ = 1.3) in two-tailed unparied *t* test. *B*, Example of p62 melting temperatures. *A, B* Based on average of two independent experiments, single measurement.

Because disulfide bond creation or reduction affects thermal stability of proteins, we applied thermal proteome profiling (25, 31) to assess the effect of DDW. Briefly, cells grown in DDW and NW medium were incubated for 3 min at 10 temperature points ranging from 37 to 67 °C and the proteins remaining soluble were quantified by nanoLC-MS/MS. For each protein, the melting temperature (T_m_) was calculated by sigmoid curve fitting, and the shift ΔT_m_ because of DDW was determined. In total, ΔT_m_ values were obtained on 3768 proteins (supplemental Table S15 and S16), of which 234 shifts reached statistical significance of *p* < 0.05 (Fig. 4*A*).

The melting curve of p62 is shown in Fig. 4*B*. DDW treatment increased its thermal stability by ΔT_m_ = 0.7 °C, in agreement with enhanced disulfide bond formation revealed in this protein by redox proteomics.

### Summary of Proteomics Results

**Fig. 5.**
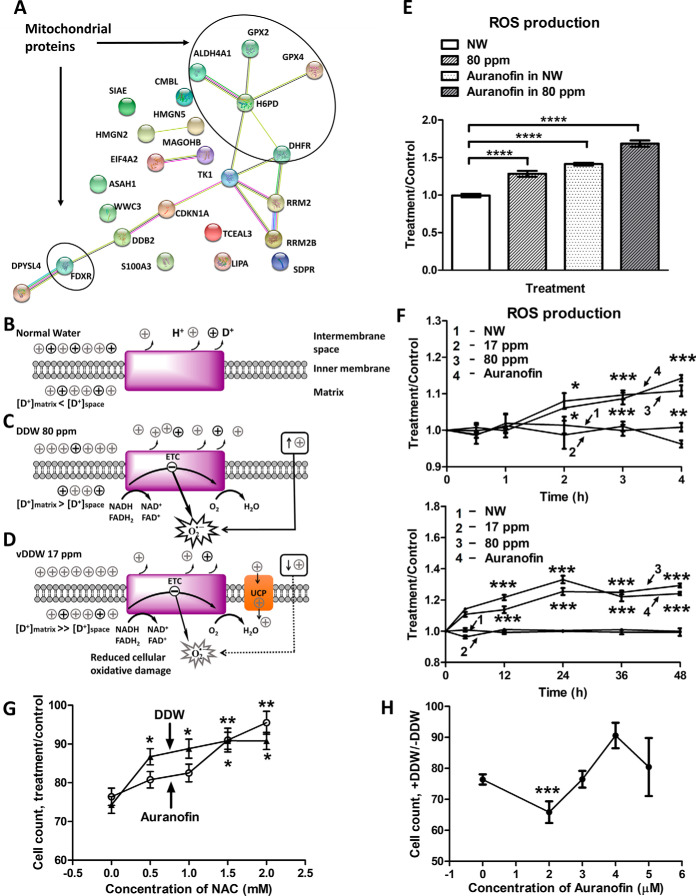
**Proposed mechanism of DDW action and its validation.** *A*, Interaction network of proteins most involved in DDW action mechanism according to summary of proteomics analysis. *B-D*, Proposed mechanism: *B*, In normal conditions, the deuterium concentration in mitochondrial matrix water is lower than in the intermembrane space water. *C*, Lowering deuterium concentration in ambient water reverses the deuterium gradient across the inner membrane. To restore the H/D equilibrium, mitochondria increase export of protons from the matrix to intermembrane space, which causes an increase in the mitochondrial membrane potential (↑+). This in turn promotes production of ROS as energy-carrying electrons are passed by ETC from NADH and FADH2 to oxygen, the final electron acceptor. Mitochondrial and cellular oxidative stress results, with slower growth and eventual apoptosis. *D*, Further lowering of deuterium concentration in ambient water causes too great an increase in ROS production to be controlled by intrinsic enzymatic antioxidant mechanisms. An additional antioxidant mechanism is triggered, in which activation by superoxide (O_2_^●−^) of UCPs combats enhanced ROS production. In this feedback model, the activated UCPs in the inner mitochondrial membrane open greater proton influx into the matrix, with concomitant reduction in the mitochondrial membrane potential (↓+), which leads to diminished O_2_^●−^ production and near restoration of the mitochondrial and cellular redox equilibrium, with reduced cellular oxidative damage. *E-H*, Validation of the DDW-induced redox disbalance mechanism. *E*, Combined effect of 80 ppm DDW with addition of 3 μm auranofin on ROS level compared with 80 ppm DDW or 3 μm auranofin alone. *F*, Time course analysis of relative ROS production in the cells treated with 3 μm auranofin, grown in vDDW and DDW (80 ppm) compared with untreated cells grown in NW. *G*, Effect on cell count of NAC addition to DDW- or auranofin-treating cells. *H*, Effect on cell count of simultaneous DDW and auranofin application compared with auranfin only. *G-H* show the mean ± SEM of nine independent experiments with four replicates, * *p* < 0.05, ** *p* < 0.01, *** *p* < 0.005, **** *p* < 0.0001 in two-tailed unparied *t* test.

The overview of FITExP, redox proteomics and TPP results is given in Table II. The proteins up- and downregulated in FITExP were analyzed by STRING separately (supplemental Table S17 and S18). 24 down regulated proteins were found to be related to oxidoreductase activity (molecular function) and glutathione metabolism (KEGG pathways). Among these molecules, 6 proteins are located in mitochondria (Fig. 5*A*), where they play important roles in ROS production by the ETC. ALDH4A1 is a mitochondrial matrix NAD-dependent dehydrogenase which catalyzes the second step of the proline degradation pathway, converting pyrroline-5-carboxylate to glutamate (59). FDXR initiates electron transport for cytochromes P450 receiving electrons from NADPH (60). Almost exclusive localization of H6PD in microsomal membranes also suggests its involvement in microsomal ETCs (61). DHFR is a member of the reductase family of enzymes that is ubiquitously expressed in all organisms. It catalyzes tetrahydrofolate regeneration by reduction of dihydrofolate using NADPH as a cofactor (62). GPX4 is a member of glutathione peroxidase family and plays a key role in protecting cells from oxidative damage by preventing membrane lipid peroxidation (63, 64). GPX2, another glutathione peroxidase, is believed to play a major role in protecting mammals from the toxicity of ingested organic hydroperoxides (65).

**Table II:** Summary of the chemical proteomics results for candidate proteins

Protein name	Gene names	Regulation	Relative oxidation DDW/Control	ΔT_m_ (^o^C)
Sequestosome-1	p62	↑	2.45	0.70
Serine/threonine-protein kinase Chk1	CHEK1	↑	/	−0.15
Cyclin-dependent kinases regulatory subunit 2	CKS2	↑	/	0.90
Gamma-aminobutyric acid receptor-associated protein-like 2	GABARAPL2	↑	/	−0.93
Neuronal pentraxin-1	NPTX1	↑	/	−1.82
TNF receptor-associated factor 1	TRAF1	↑	/	−0.33
Calcium/calmodulin-dependent protein kinase type 1	CAMK1	↑	/	−0.40
Iron-responsive element-binding protein 2	IREB2	↑	/	−1.06
MORF4 family-associated protein 1	MRFAP1	↑	/	0.58
Low-density lipoprotein receptor	LDLR	↑	1.02	0.80
Kunitz-type protease inhibitor 2	SPINT2	↑	1.33	/
Kinesin-like protein KIF23	KIF23	↑	1.52	1.48
Hydroxymethylglutaryl-CoA synthase, cytoplasmic	HMGCS1	↑	3.64	0.58
Calcium-binding and coiled-coil domain-containing protein 2	CALCOCO2	↑	/	−0.23
Uncharacterized protein KIAA1143	KIAA1143	↑	/	−0.01
Histone H2AX	H2AFX	↑	1.69	/
POTE ankyrin domain family member E	POTEE	↑	1.38	/
T-complex protein 11-like protein 2	TCP11L2	↑	/	0.25
Cell division cycle protein 20 homolog	CDC20	↑	/	−0.56
Heme oxygenase 1	HMOX1	↑	/	0.10
Phospholipid hydroperoxide glutathione peroxidase, mitochondrial	GPX4	↓	/	1.08
Delta-1-pyrroline-5-carboxylate dehydrogenase, mitochondrial	ALDH4A1	↓	/	−0.01
NADPH:adrenodoxin oxidoreductase, mitochondrial	FDXR	↓	1.66	−0.19
Ribonucleoside-diphosphate reductase subunit M2 B	RRM2B	↓	/	−0.21
Non-histone chromosomal protein HMG-17	HMGN2	↓	/	2.04
Serum deprivation-response protein	SDPR	↓	/	0.57
Cyclin-dependent kinase inhibitor 1	CDKN1A	↓	/	−0.54
Lysosomal acid lipase/cholesteryl ester hydrolase	LIPA	↓	/	1.57
Carboxymethylenebutenolidase homolog	CMBL	↓	0.85	0.44
Dihydropyrimidinase-related protein 4	DPYSL4	↓	/	0.43
Sialate O-acetylesterase	SIAE	↓	/	−4.93
Eukaryotic initiation factor 4A-II	EIF4A2	↓	/	−0.71
DNA damage-binding protein 2	DDB2	↓	/	−0.13
Ribonucleoside-diphosphate reductase subunit M2	RRM2	↓	/	−0.21
High mobility group nucleosome-binding domain-containing protein 5	HMGN5	↓	/	−0.08
Glucose 1-dehydrogenase	H6PD	↓	/	−1.10
Dihydrofolate reductase	DHFR	↓	/	−0.14
Protein mago nashi homolog 2	MAGOHB	↓	/	0.89
Thymidine kinase, cytosolic	TK1	↓	/	−0.57
Protein S100-A3	S100A3	↓	/	0.92
Acid ceramidase;Acid ceramidase subunit alpha; Acid ceramidase subunit beta	ASAH1	↓	/	−0.03
Glutathione peroxidase 2	GPX2	↓	/	−1.17
Protein WWC3	WWC3	↓	/	−1.51
Transcription elongation factor A protein-like 3	TCEAL3	↓	/	−0.84

“↑” means the protein is up-regulated (“↓” - down-regulated) in DDW specifically in respect to control drugs and NW. “/” means the protein was not identified in redox proteomics or TPP analysis.

### Mechanism Validation

In order to test the redox disbalance hypothesis, the fluorescent reporter DCF-DA was employed to measure ROS production in cells growing at different conditions. Auranofin, that elicits strong cytotoxicity in tumor cells through overproduction of ROS, was used as a control drug. The production of ROS in the cells grown in 80 ppm DDW for 48 h was elevated compared with those grown in NW, as expected (Fig. 5*F*). Also, application of auranofin to 80 ppm DDW-grown cells increased the ROS level very significantly compared with the cells either treated with auranofin or 80 ppm DDW-grown (Fig. 5*E*). In 80 ppm DDW, the cellular ROS levels increased compared with NW already after 2 h of growth, as it did in auronafin treatment, whereas in the cells grown in vDDW these levels did not change significantly (Fig. 5*F*).

In addition, we attempted to counter the DDW toxic effect by simultaneous addition of NAC, a thiol-reducing antioxidant agent. For both DDW and auranofin treatments, the suppressed cell proliferation was largely restored at higher NAC concentrations (Fig. 5*G*, supplemental Fig. S4*A*). This result supports the notion that DDW leads to overproduction of cellular ROS, like auranofin.

In simultaneous growth in DDW and application of auranofin, the biggest synergistic suppression of cell growth (-30% compared with auranofin only) occurred at a relatively low auranofin concentration, corresponding to ≈0.5 IC_50_ for auranofin (Fig. 5*H*, supplemental Fig. S4*B*). Therefore, DDW may indeed be useful as an adjuvant to conventional anticancer treatment. Simultaneous DDW intake may even allow for lowering the concentration of chemotherapeutics administered to patients and thus for reducing the overall toxicity burden and concomitant side effects.

## DISCUSSION

### Proposed Mechanism

Based on the above results, we hypothesize that DDW affects mitochondrial membrane potential and thereby creates a disbalance between cellular oxidation and reduction. This is also consistent with what have been reported in several studies (9, 18, 19). In normal conditions, the deuterium concentration in mitochondrial matrix water is lower than in the intermembrane space water, creating a positive deuterium gradient across the inner membrane (Fig. 5*B*). This is because of the mainly metabolic origin of the matrix water, and the lower natural deuterium content in the prime sources of metabolic water, organic fats (66). Lowering the deuterium concentration in ambient water reverses the deuterium gradient across the inner membrane (Fig. 5*C*). This triggers as yet unknown mechanism that increases the export of protons from the matrix to intermembrane space and thus increases the mitochondrial membrane potential (↑+). Such an increase in turn promotes ROS production as energy-carrying electrons pass by ETC from NADH and FADH_2_ to oxygen, the final electron acceptor. Mitochondrial and cellular oxidative stress results, which is partially countered by intrinsic enzymatic antioxidant mechanisms, such as those involving superoxide dismutases 1 and 2 or catalases, but with slower growth and eventual apoptosis of some cells as a net outcome. However, further lowering of deuterium concentration (vDDW, 17 ppm) in ambient water causes too great an increase in ROS production to be controlled by intrinsic enzymatic antioxidant mechanisms. Previously (67), an additional antioxidant mechanism has been suggested, in which an activation by superoxide (O_2_^●−^) of UCPs combats the enhanced ROS production. In a similar feedback model (Fig. 5*D*), the activated UCPs in the inner mitochondrial membrane open greater proton influx into the matrix, with concomitant reduction in the mitochondrial membrane potential (↓+), which leads to diminished O_2_^●−^ production and near restoration of the mitochondrial and cellular redox equilibrium. This hypothetical mechanism explains both the suppression of cell proliferation by medium (80–100 ppm D) deuterium depletion as well as the restoration of normal growth at extreme (<20 ppm) deuterium depletions.

## CONCLUSIONS

Our experiments confirmed the effect of deuterium depletion on cancer cell growth. The novel aspect is the suggested molecular mechanism supporting the anticancer effect of DDW. Our results indicate that DDW inhibits cell proliferation mainly through causing a disbalance between ROS production and neutralization in mitochondria, and thus inducing oxidative stress in the cells. DDW also modulates the expression of proteins involved in such cell-wide processes as cell cycle, oxidoreductase activity, p53 signaling pathway, glutathione metabolism, etc. The proposed model explaining the bulk of observations suggests that, when the concentration of deuterium in the media becomes lower than inside the mitochondria, the mitochondrial membrane potential increases, which enhances ROS production and leads to cell growth suppression. However, when DDW concentration becomes even lower, the feedback loop is activated and the ROS balance becomes restored. The time scale of DDW action also hinted that DDW may arrest or prolong late S and G2-phase in A549 cells; however, testing this hypothesis experimentally was outside the scope of the current study. Summarizing, DDW seems to have potential in antitumor therapy, especially in the modalities inducing oxidative stress in cancer cells.

## DATA AVAILABILITY

Excel files containing the analyzed data are provided in Supplementary Materials. The mass spectrometry proteomics data have been deposited to the ProteomeXchange Consortium (http://proteomecentral.proteomexchange.org) via the PRIDE partner repository with the dataset identifier PXD011085.

## Supplementary Material

Anticancer effect of deuterium depleted water - redox disbalance leads to oxidative stres

Supplementary Table S1

Supplementary Table S2

Supplementary Table S5

Supplementry Table S6

Supplementry Table S7

Supplementry Table S8

Supplementry Table S9

Supplementry Table S10

Supplementry Table S13

Supplementry Table S14

Supplementry Table S15

Supplementry Table S16

## Footnotes

^1^ The abbreviations used are:

DDW: deuterium depleted water
AGC: automatic gain control
ACN: acetonitrile
CAMP: camptothecin
CE: collision energy
CV: coefficient of variation
DCF-DA: 2′, 7′-dichlorodihydrofluorescein diacetate
DEME: Dulbecco's Modified Eagle's Medium
DTT: 1,4-dithiothreitol
EDTA: ethylenediaminetetraacetic
ETC: eletron transpaort chain
FA: formic acid
FBS: fetal bovine serum
FDR: false discovery rate
FITExP: Functional Identification of Targets by Expression Proteomics
HCD: higher-energy collision dissociation
IAA: iodoacetamide
MTT: 3-(4,5-dimethylthiazol-2-yl)-2,5-diphenyltetrazolium bromide
MTX: methotrexate
NAC: N-acetyl cysteine
NCE: normalized collision energy
nanoLC-MS/MS: nanoflow-high-performance liquid chromatography–tandem mass spectrometry
NW: normal water
OPLS-DA: Orthogonal Projections to Latent Structures Discriminant Analysis
PCA: principal component analysis
PCTL: paclitaxel
ROS: reactive oxygen species
RT: room temperature
SEM: standard error of the mean
TCEP: Tris-(2-Carboxyethyl)phosphine, hydrochloride
TPP: thermal proteome profling
UCPs: uncoupling proteins.
