# Supplementary material for: Anticancer Effect of Deuterium Depleted Water - Redox Disbalance Leads to Oxidative Stress
Source: Mol Cell Proteomics. 2019 Sep 13;18(12):2373–87. doi: 10.1074/mcp.RA119.001455 (PMC6885702; doi:10.1074/mcp.RA119.001455)
Supplement: Anticancer effect of deuterium depleted water - redox disbalance leads to oxidative stres [file 144400_2_supp_382929_pwmgxf.pdf]

**Anticancer effect of deuterium depleted water - redox disbalance leads to oxidative stress**

Xuepei Zhang, Massimiliano Gaetani, Alexey Chernobrovkin, Roman A. Zubarev*

***Supplementary Figure captions:***

**Table S1.** Common identified proteins in protein abundances analysis.

**Table S2.** Top 50 up and down regulated proteins specific for DDW and the abundance ratios to control.

**Table S3.** STRING analysis result of top 50 up regulated proteins

**Table S4.** STRING analysis result of top 50 down regulated proteins

**Table S5.** Result of time course analysis.

**Table S6.** Time course analysis of proteins most regulated by MTX.

**Table S7.** Time course analysis of proteins most regulated by DDW.

**Table S8.** Time course analysis of proteins most regulated by PCTL.

**Table S9.** Common proteins identified in redox proteomics result.

**Table S10.** Common peptides identified in redox proteomics result.

**Table S11.** Top 20 oxidized and reduced proteins.

**Table S12.** Oxidation levels of p62 peptides in DDW vs NW.

**Table S13.** Common proteins in cells growing in NW or DDW medium in redox result.

**Table S14.** Common peptides in cells growing in NW or DDW medium in redox result.

**Table S15.** Result of TPP analysis.

**Table S16.** R analysis of TPP result.

**Table S17.** STRING analysis result of overlap top 50 up regulated proteins with disbalance in oxidation -reduction level or changes in stability.

**Table S18.** STRING analysis result of top 50 down regulated proteins with disbalance in oxidation-reduction level or changes in stability.

**Figure. S1.** Survival of A549 cell line grown in media with different deuterium concentrations from 17-150 ppm.

**Figure. S2.** DDW effect on cell proliferation and dynamic analysis of regulation of DDW on proteome abundance.

**Figure. S3.** Inhibitory curve of auranofin.

**Figure. S4.** Validation of DDW anticancer mechanism.

**Table S3. STRING analysis result of top 50 up regulated proteins**

| | Pathway ID | Pathway description | Observed gene count | False discovery rate |
|---|---|---|---|---|
| Cellular Component (GO) | GO:0005776 | autophagosome | 4 | 0.02 |
| | GO:0044754 | autolysosome | 2 | 0.0407 |
| UniProt Keywords | KW-0131 | Cell cycle | 9 | 0.0047 |
| | KW-0832 | Ubl conjugation | 16 | 0.0135 |

## Table S4. STRING analysis result of top 50 down regulated proteins

| | Pathway ID | Pathway description | Observed gene count | False discovery rate |
|---|---|---|---|---|
| Biological Process (GO) | GO.0009165 | nucleotide biosynthetic process | 6 | 0.0325 |
| | GO.0010224 | response to UV-B | 3 | 0.0325 |
| Molecular Function (GO) | GO.0004748 | ribonucleoside-diphosphate reductase activity, thioredoxin disulfide as acceptor | 2 | 0.0183 |
| | GO.0016491 | oxidoreductase activity | 9 | 0.0183 |
| Cellular Component (GO) | GO.0005829 | cytosol | 20 | 0.0122 |
| KEGG Pathways | 4115 | p53 signaling pathway | 6 | 3.22E-06 |
| | 480 | Glutathione metabolism | 4 | 0.000816 |
| | 1100 | Metabolic pathways | 10 | 0.0242 |
| | 5161 | Hepatitis B | 4 | 0.0242 |
| | 5214 | Glioma | 3 | 0.0242 |

**Table S11. Top 20 oxidized and reduced proteins.** OX - oxdiation percentage, C – NW, A – auronafin, D – DDW.

| Oxidized or reduced | Gene names | Oxidation percentage (OX) | | | | | | | | | |
|---|---|---|---|---|---|---|---|---|---|---|---|
| | | A-OX-1 | A-OX-2 | A-OX-3 | C-OX-1 | C-OX-2 | C-OX-3 | D-OX-1 | D-OX-2 | D-OX-3 | Rank |
| Oxidized | HNRNPA1 | 0.4 | 0.6 | 2.8 | 3.0 | 3.6 | 2.5 | 11.2 | 11.5 | 11.0 | 1 |
| | RPAP3 | 2.2 | 3.2 | 5.3 | 12.1 | 4.9 | 12.1 | 42.3 | 33.6 | 42.9 | 2 |
| | p62 | 13.0 | 11.7 | 10.8 | 16.1 | 18.5 | 19.7 | 34.5 | 35.7 | 34.5 | 3 |
| | RPL32 | 1.5 | 7.7 | 6.4 | 8.2 | 4.4 | 3.5 | 30.8 | 20.7 | 28.5 | 4 |
| | CA12 | 57.7 | 60.2 | 57.3 | 65.4 | 61.8 | 61.6 | 76.3 | 75.2 | 77.0 | 5 |
| | DDX39B | 3.2 | 3.3 | 4.9 | 4.0 | 3.7 | 5.1 | 8.5 | 7.4 | 8.3 | 6 |
| | PACSIN2 | 16.8 | 12.8 | 14.1 | 16.5 | 14.4 | 9.9 | 25.8 | 27.2 | 24.9 | 7 |
| | ANXA2 | 2.7 | 1.5 | 2.4 | 5.1 | 5.9 | 5.7 | 12.1 | 12.2 | 13.6 | 8 |
| | FKBP4 | 6.0 | 6.2 | 6.8 | 7.0 | 8.0 | 7.4 | 16.5 | 23.7 | 16.3 | 9 |
| | PSMD4 | 4.1 | 9.5 | 7.1 | 2.0 | 6.2 | 10.2 | 19.3 | 20.1 | 22.1 | 10 |
| | RFC2 | 9.1 | 9.3 | 14.9 | 10.0 | 6.0 | 13.6 | 24.9 | 31.0 | 25.0 | 11 |
| | SARS2 | 1.2 | 2.3 | 4.2 | 1.2 | 1.8 | 1.9 | 13.3 | 17.5 | 9.5 | 12 |
| | MYO1B | 7.2 | 8.2 | 7.9 | 7.4 | 9.5 | 9.6 | 15.0 | 12.8 | 16.8 | 13 |
| | ACTR2 | 8.7 | 5.5 | 5.2 | 4.2 | 6.1 | 10.9 | 24.1 | 16.5 | 24.1 | 14 |
| | HSPA9 | 4.6 | 6.3 | 9.1 | 12.6 | 7.0 | 7.4 | 19.5 | 17.6 | 21.9 | 15 |
| | FSCN1 | 3.5 | 6.0 | 6.1 | 10.5 | 10.0 | 11.7 | 21.5 | 19.2 | 23.0 | 16 |
| | GSK3A | 4.9 | 2.3 | 8.1 | 10.7 | 8.0 | 9.5 | 25.8 | 17.9 | 26.2 | 17 |
| | CTPS1 | 7.1 | 7.9 | 8.4 | 7.8 | 7.1 | 7.7 | 15.4 | 12.0 | 11.4 | 18 |
| | TIPRL | 4.3 | 6.5 | 6.9 | 6.4 | 9.5 | 6.0 | 15.9 | 12.6 | 13.1 | 19 |
| | XPO5 | 7.6 | 9.6 | 10.8 | 1.7 | 3.8 | 5.8 | 30.1 | 18.3 | 27.8 | 20 |
| Reduced | ITGAV | 67.9 | 68.2 | 67.4 | 72.1 | 69.0 | 72.0 | 51.6 | 59.3 | 53.7 | 1 |
| | CELF1 | 23.2 | 14.8 | 25.5 | 25.9 | 22.9 | 26.8 | 5.6 | 8.8 | 6.8 | 2 |
| | GLG1 | 69.5 | 66.9 | 74.3 | 57.8 | 59.9 | 63.3 | 45.3 | 43.4 | 43.2 | 3 |
| | EIF4G2 | 15.0 | 14.3 | 16.6 | 7.9 | 18.2 | 17.5 | 4.5 | 2.7 | 3.6 | 4 |
| | JAG1 | 84.3 | 83.3 | 94.9 | 74.0 | 80.8 | 79.7 | 61.8 | 36.6 | 62.2 | 5 |
| | UBE2M | 11.4 | 17.6 | 16.8 | 10.9 | 11.1 | 15.3 | 3.9 | 2.4 | 8.1 | 6 |
| | TSPAN14 | 74.7 | 73.6 | 82.3 | 63.6 | 42.2 | 48.2 | 30.9 | 24.2 | 26.7 | 7 |
| | EWSR1 | 9.8 | 7.3 | 12.9 | 7.4 | 8.8 | 8.6 | 3.7 | 2.4 | 5.9 | 8 |
| | EPHA2 | 73.8 | 75.8 | 76.4 | 65.5 | 66.4 | 66.3 | 53.9 | 63.0 | 54.9 | 9 |
| | DIS3 | 16.8 | 26.7 | 22.3 | 18.6 | 19.2 | 21.3 | 13.2 | 7.3 | 14.2 | 10 |
| | ASNS | 23.0 | 22.5 | 26.6 | 11.4 | 16.8 | 12.1 | 4.9 | 2.5 | 7.3 | 11 |
| | HNRNPF | 6.8 | 10.0 | 16.8 | 16.0 | 16.9 | 17.3 | 2.3 | 5.4 | 4.8 | 12 |
| | SMC1A | 20.0 | 22.7 | 23.5 | 15.5 | 12.5 | 13.6 | 8.7 | 7.3 | 7.4 | 13 |
| | BSG | 51.0 | 51.0 | 46.0 | 78.1 | 80.7 | 87.0 | 23.5 | 18.4 | 35.5 | 14 |
| | ALCAM | 79.9 | 78.6 | 87.2 | 35.9 | 49.3 | 53.9 | 22.6 | 20.1 | 23.8 | 15 |
| | LMAN2 | 48.5 | 44.1 | 53.2 | 40.9 | 44.4 | 41.3 | 38.6 | 28.2 | 35.7 | 16 |
| | RPL36A | 7.3 | 7.1 | 8.2 | 4.8 | 6.9 | 8.2 | 4.7 | 3.0 | 5.2 | 17 |
| | DDB1 | 6.7 | 9.3 | 6.3 | 8.7 | 5.5 | 7.9 | 4.0 | 3.1 | 5.5 | 18 |
| | ERGIC3 | 76.2 | 78.2 | 81.0 | 48.6 | 53.2 | 49.4 | 35.8 | 33.1 | 37.1 | 19 |
| | RPS15A | 10.5 | 8.9 | 15.1 | 13.1 | 12.1 | 11.6 | 8.1 | 6.0 | 8.8 | 20 |

## Table S12. Oxidation levels of p62 peptides in DDW vs NW.

| Sequence | Mass | Charges | Score | Peptide number | | Oxidation percentage (%) | | | | | | |
|---|---|---|---|---|---|---|---|---|---|---|---|---|
| | | | | NW | DDW | NW-1 | NW-2 | NW-3 | DDW-1 | DDW-2 | DDW-3 | p value |
| 23-46 | 2453.99 | 5;6 | 114.63 | 1 | 1 | 13.53 | 9.45 | 11.73 | 18.83 | 17.62 | 18.51 | 0.005446 |
| 119-139 | 2080.96 | 5 | 94.632 | 1 | 1 | 21.79 | 31.26 | 30.06 | 56.40 | 56.32 | 54.89 | 0.000732 |
| 151-166 | 1719.66 | 5;6 | 96.208 | 1 | 1 | 15.99 | 21.19 | 22.08 | 42.50 | 41.99 | 45.63 | 0.000437 |

**Table S17. STRING analysis result of overlap top 50 up regulated proteins with disbalance in oxidation -reduction level or changes in stability**

| | Pathway ID | Pathway description | Observed gene count | False discovery rate |
|---|---|---|---|---|
| KEGG Pathways | 5203 | Viral carcinogenesis | 4 | 0.00738 |

**Table S18. STRING analysis result of top 50 down regulated proteins with disbalance in oxidation-reduction level or changes in stability**

| | Pathway ID | Pathway description | Observed gene count | False discovery rate |
|---|---|---|---|---|
| Molecular Function (GO) | GO.0016491 | oxidoreductase activity | 8 | 0.00167 |
| | GO.0004748 | ribonucleoside-diphosphate reductase activity, thioredoxin disulfide as acceptor | 2 | 0.00407 |
| | GO.0003824 | catalytic activity | 16 | 0.00774 |
| KEGG Pathways | 480 | Glutathione metabolism | 4 | 8.55E-05 |
| | 4115 | p53 signaling pathway | 4 | 0.000144 |
| | 1100 | Metabolic pathways | 8 | 0.00326 |
| | 240 | Pyrimidine metabolism | 3 | 0.0153 |

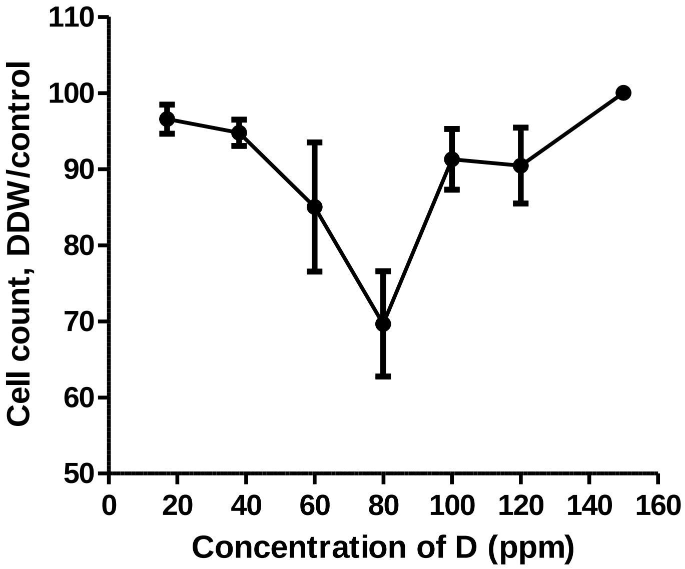


**FIG. S1.** Survival of A549 cell line grown in media with different deuterium concentrations from 17-150 ppm

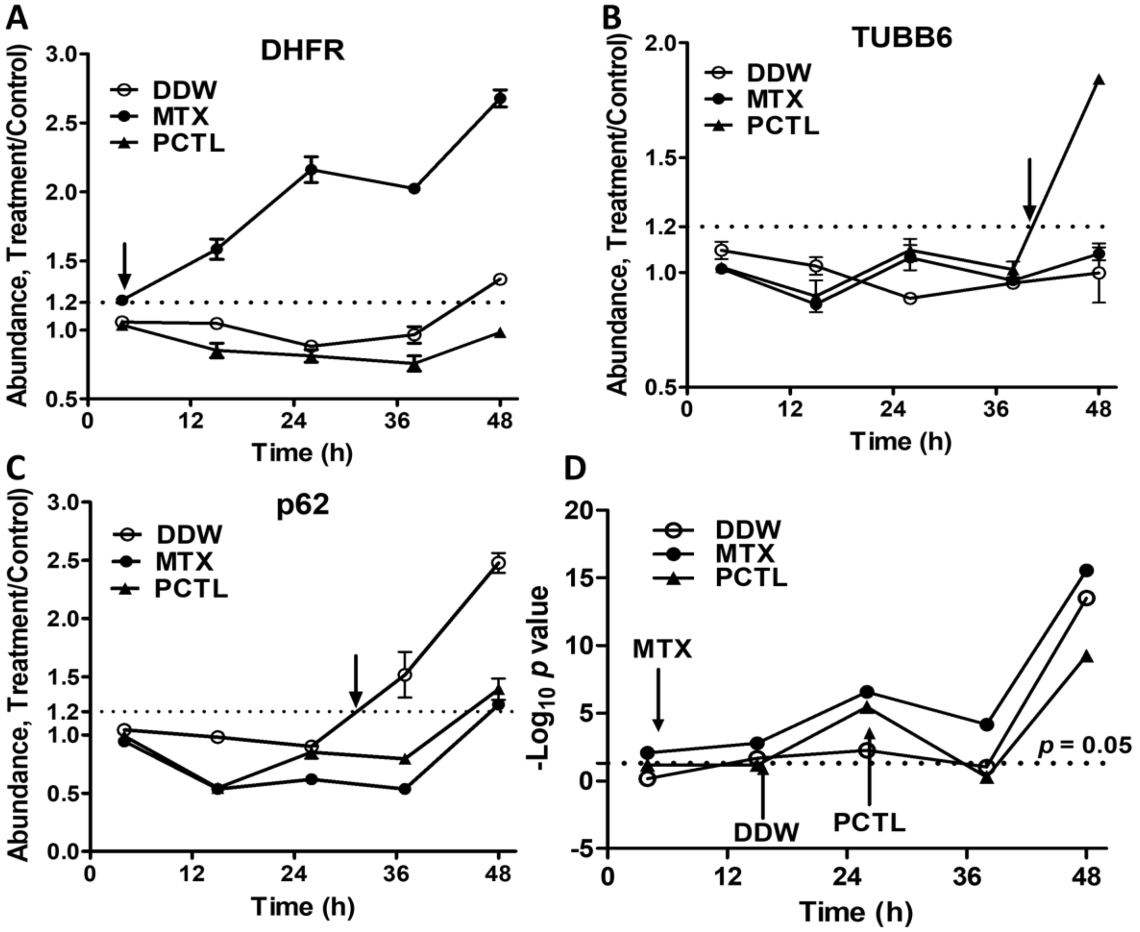


**FIG. S2.** DDW effect on cell proliferation and dynamic analysis of regulation of DDW on proteome abundance. Abundance dynamic changes of DHFR (**A**), TUBB6 (**B** and p62 (**C**). **D** Statistical significance analysis of top 50 up and down regulated proteins in FITExP for each treatment at each time point. Error bars represent SEM, n = 2.

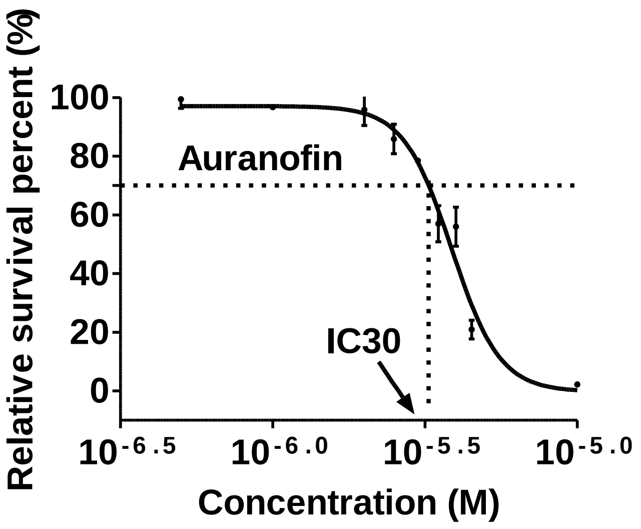


**FIG. S3.** Inhibitory curve of auranofin using Prism following logarithmic transformation of concentration and fitting curve using equation of log(inhibitor) vs. response – Variable slop (four parameters). Error bars represent SEM, n = 3.

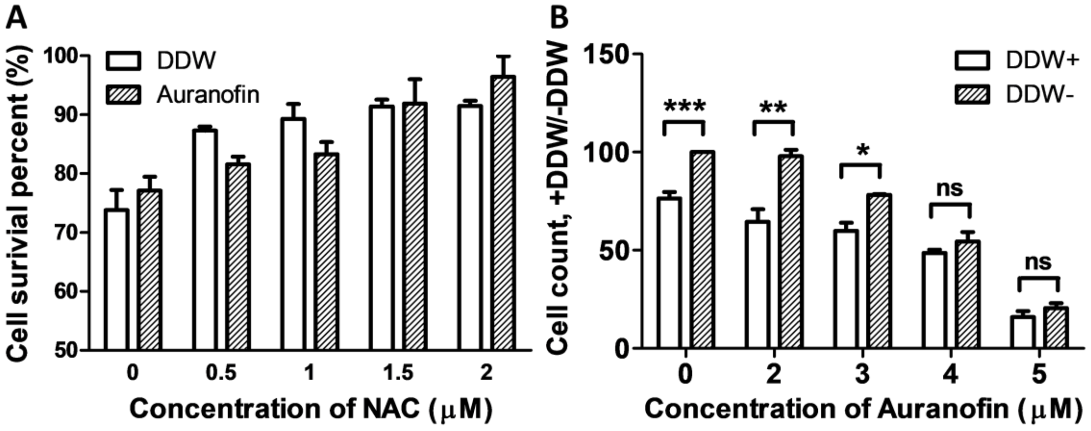


**FIG. S4. Validation of DDW anticancer mechanism.** *A*, Combined effect of DDW and Auranofin on cell survival. *B*, Effect of DDW and Auranofin on cell survival in prensence of NAC. Error bars represent SEM with triplcates measurements, ns P>0.05, *P<0.05, **P<0.01, ***P<0.005 (two-tailed unparied t-tests).
